# Combined Effects of Nano-Polystyrene and Heavy Metal Mixture on the Bioaccumulation of Heavy Metals and Physiological Changes in *Macrobrachium rosenbergii*

**DOI:** 10.3390/jox15040113

**Published:** 2025-07-07

**Authors:** Mahdi Banaee, Amir Zeidi, Amal Beitsayah, Cristiana Roberta Multisanti, Caterina Faggio

**Affiliations:** 1 Department of Fishery Sciences, Faculty of Natural Resources, Behbahan Khatam Alanbia University of Technology, Behbahan 63973-63616, Iran; zeidi.amir@gmail.com; 2 Aquaculture Department, Faculty of Fisheries and Environmental Sciences, Gorgan University of Agriculture Sciences and Natural Resources, Gorgan 81746-73441, Iran; amal_beitsayah@yahoo.com; 3 Department of Veterinary Sciences, University of Messina, Viale Giovanni Palatucci, snc, 98168 Messina, Italy; 4 Department of Chemical, Biological, Pharmaceutical and Environmental Sciences, University of Messina, 98122 Messina, Italy; cfaggio@unime.it; 5 Department of Eco-sustainable Marine Biotechnology, Stazione Zoologica Anton Dohrn, 80121 Naples, Italy

**Keywords:** *Macrobrachium rosenbergii*, nano-plastics, Raman spectroscopy, heavy metals, lipid peroxidation

## Abstract

Contaminants such as nano-polystyrenes (NPs) and heavy metal cocktail (HMC) have been found to disrupt physiological functions in aquatic organisms. Although HMC and NPs alone induce oxidative stress, their combined effects are not well understood. This study aimed to assess the combined effects of HMC and NPs on the freshwater shrimp (*Macrobrachium rosenbergii*). Shrimp were divided into seven groups, including the control group, and the experimental groups co-exposed to 0, 50, 100, 150, 200, and 250 µg/L NPs combined with 0.5 mg/L HMC. After 14 days, shrimp were sampled, and their hepatopancreas and muscle tissues were analyzed for oxidative biomarkers, biochemical parameters, and metabolic profiles. Moreover, the bioaccumulation rate of heavy metals was measured. Results showed that co-exposure to NPs and HMC increased superoxide dismutase, glutathione peroxidase, glutathione reductase activities, and malondialdehyde levels, while reducing glutathione and total antioxidant capacity. The integrated biomarker response indicated that co-exposure to HMC and NPs induces oxidative stress. A significant decrease was observed in aspartate aminotransferase, gamma-glutamyl transpeptidase, and alkaline phosphatase activities, glycogen, triglyceride, and total protein levels. However, lactate dehydrogenase activity was significantly increased. Co-exposure to HMC and NPs increased heavy metal bioaccumulation, induced oxidative stress, biochemical changes, and enhanced HMC toxicity in shrimp.

## 1. Introduction

Contamination of aquatic ecosystems with nanoplastics (NPs), along with their bioavailability and associated risks, is an emerging concern in marine and oceanic environments [1,2]. Studies indicate that the transport of nanoplastics in seawater-saturated sand is influenced by surface charge and functional groups. Negatively charged particles, such as those with carboxylic or sulfonic groups, exhibit increased mobility, particularly in the presence of humic acid or under lower salinity conditions [3]. Although analytical limitations still hinder precise quantification of nanoplastics in natural waters, studies have detected nano-polystyrene particles in surface and drinking waters at concentrations up to several tens of µg/L, supporting the environmental relevance of the exposure levels adopted in this study. Indeed, Vega-Herrera et al. [4] demonstrated that micro- and nanoplastics (MNPLs, 700 nm to 20 μm) are present even in bottled drinking water. Moreover, over twenty-eight different additives, including phthalates, were detected alongside MNPLs in these samples [5]. Schirinzi et al. [6] developed a sensitive LC-HRMS method with APPI for detecting nano-polystyrene in surface waters. Scientific evidence suggests that polystyrene, is the third most found type of plastic polymer in atmospheric fallout. In general, plastic polymers are subject to continuous exposure to environmental agents. Specifically, wind and wave action, mechanical action, and ultraviolet (UV) light, through photo-oxidation, as well as microbiological degradation processes, cause the breakdown of plastic waste into smaller units, i.e., microplastics (<5 mm) and, after further fragmentation, nanoplastics (≤0.1 µm). These particles are characterized by their environmental persistence leading them able to easily interact with biological systems [2,7]. Due to its unique properties, nano-polystyrenes (NPs) plays an essential role in various industries, including pharmaceuticals, packaging, paints, electronics, and cosmetics [8,9]. NPs are also used in water purification and pollutant removal [10] and in the research and behavior of nanomaterials in biological and environmental systems. Scientific evidence suggests that polystyrene is the third most found type of plastic polymer in atmospheric fallout [11,12] and it is also one of the most abundant in aquatic ecosystems [13]. Studies showed that NPs could induce hepatotoxicity [14,15,16], intestinal barrier dysfunction [17], and neurotoxicity [18]. Moreover, cellular studies displayed mitochondrial damage, autophagy disruption, and apoptosis [19,20]. Sadeghinia et al. [21] found that NPs activated oxidative stress and apoptosis in cancer and fibroblast cells. Recent studies have also reported NP toxicity in aquatic organisms. For instance, Qiao et al. [22] found that NPs exhibited more toxic effects to aquatic invertebrates than micro-polystyrene particles. NP exposure reduced survival, growth, and reproduction in *Daphnia pulex* [23] and induced oxidative stress and nervous system disruptions in the species *Litopenaeus vannamei* [24]. Moreover, DNA damage and strand breaks were observed in *Dreissena polymorpha* [8]. Sökmen et al. [25] demonstrated neurotoxicity in zebrafish embryos, showing NPS accumulation in the brain and increased ROS levels. The bioaccumulation of NPs was reported in *Corbicula fluminea* [26]. Lei et al. [27] observed size-dependent toxicity in *Caenorhabditis elegans*, with nanoparticles causing severe neurotoxicity and oxidative stress. Plastic debris, microplastics, and nanoplastics are newly identified waterborne contaminants with the potential to impact biological and non-biological pollutants within water ecosystems [28]. Research has demonstrated that plastics can act as *Trojan horses*, thereby facilitating the translocation of other xenobiotics into food webs. Furthermore, plastics have been demonstrated to enhance the bioavailability and toxicity of chemical and biological pollutants, or, in contrast, may antagonistically affect medicine and drugs [29,30].

In this regard, studies showed that interaction between NPs and heavy metals could significantly affect the behavior, bioavailability, and toxicity of heavy metals in aquatic ecosystems. Also, this interaction can enhance the mobility and persistence of heavy metals in water. Che et al. [31] reported increased lipid peroxidation and decreased antioxidant enzyme activity in *Eriocheir sinensis* exposed to NPs and cadmium (Cd). Antioxidant response alterations were observed in *Tachypleus tridentatus* co-exposed to NPs and copper (Cu^2+^) [32]. Similarly, Luo et al. [33] showed that NPs increased Cd bioaccumulation in *Bellamya aeruginosa*. In addition to the onset of oxidative stress, NPs and Cd exposure have been found to be able to disrupt the gut microbiome in *E. sinensis*, increasing pathogenic *Spiroplasma* abundance [31].

Heavy metals are well-known aquatic contaminants that can bioaccumulate in invertebrates and exert a variety of ecotoxicological effects, including oxidative damage, metabolic disruption, and impaired immune and reproductive functions. Their toxicity often depends on environmental concentrations, conditions, and their chemical speciation in water. Nano-polystyrene particles, due to their high surface-area-to-volume ratio and strong negative surface charge, can adsorb metal ions through electrostatic interactions, hydrogen bonding, and surface complexation. This property enhances the mobility, persistence, and bioavailability of metals in aquatic systems. Therefore, this study hypothesized that exposure to NPs and a mixture of heavy metals would significantly affect freshwater shrimp (*Macrobrachium rosenbergii*) health. The heavy metal cocktail (HMC) included ten metals commonly found in polluted freshwater ecosystems (Fe, Zn, Cd, Ni, Pb, Mg, Mn, V, Co, and Cu), each at 0.05 mg/L, for a total concentration of 0.5 mg/L, chosen to simulate a sub-lethal yet ecologically relevant exposure scenario. *M. rosenbergii* is highly sensitive to various environmental pollutants due to its filter-feeding behavior, permeable exoskeleton, and aquatic respiration, and it readily accumulates toxic substances from water and sediments. Moreover, it is a widely used freshwater invertebrate model in ecotoxicology. It is a decapod crustacean with a life cycle that includes larval development in brackish water and maturation in freshwater. Adults can reach 25–30 cm, although in this study, shrimps with an average weight of 10.5 ± 2 g were used. They are benthic, omnivorous, and highly sensitive to waterborne contaminants due to their permeable exoskeleton and filter-feeding behavior. These characteristics make *M. rosenbergii* a suitable bioindicator species for studying pollutant bioaccumulation and sub-lethal physiological effects [34]. Therefore, this study aimed to evaluate the effects of NPs in combination with a heavy metal cocktail on the bioavailability of heavy metals, oxidative stress induction, and alterations in the biochemical profile of shrimp tissues.

## 2. Materials and Methods

### 2.1. Collection and Acclimation of Shrimps

Freshwater shrimps (*Macrobrachium rosenbergii*, average weight: 10.5 ± 2 g) were collected from a local fish farm (Ahvaz, Khuzestan Province, Iran). The shrimps were rapidly transferred to the aquaculture laboratory at the Natural Resources Faculty (Behbahan Khatam Alanbia University of Technology, Iran) and located into a 1000 L fiberglass tank. Specimens were acclimated for two weeks in aerated freshwater (23 ± 2 °C, pH: 7.4 ± 0.2, and dissolved oxygen: 6.5 ± 0.5 mg/L under a 14:10 h light–dark cycle) and were fed a commercial diet (Beyza feed Mill, Shiraz, Iran) and dried *Spirulina* twice daily.

### 2.2. Chemicals

#### 2.2.1. Preparation of the Heavy Metal Cocktail

To prepare a final concentration of 50 mg/L for a heavy metal cocktail (HMC) containing 10 different metals, each stock solution was at 5 mg/L. The final total concentration of the HMC was 0.5 mg/L, prepared by adding 0.05 mg/L of each of the 10 selected metal salts. Equal volumes of the following 10 metal solutions were used: iron (III) chloride, zinc oxide, cadmium chloride, nickel nitrate, lead acetate, magnesium sulfate, manganese sulfate, vanadium (III) chloride, cobalt nitrate, and copper (II) sulfate. All metals were obtained from Merck Co. (Laway, NJ, USA).

#### 2.2.2. Nano-Polystyrene Particles

Commercial nano-polystyrene (NP) suspension (as solution, 0.075 g in 1 mL) was purchased from Tehran Paint Research Institute, Iran (size: 100 nm; Dynamic Light Scattering (DLS): ~50 nm; zeta potential: −160 mV).

### 2.3. Preparation of Experimental Solutions

NP suspensions were prepared by dispersing NPs particles in distilled water and placed in an ultrasonic device (XUBA3) (Grant Instruments (Cambridge) Ltd., Royston, UK) for 10 min to achieve uniform distribution. Heavy metal cocktails (HMCs) were prepared by mixing equal concentrations of selected heavy metals to achieve a final concentration of 0.5 mg/L. The experimental solutions were renewed every 24 h to maintain exposure stability.

### 2.4. Experimental Design

A total of 420 shrimps were randomly assigned to seven experimental groups, each with three replicates. Specifically, each experimental group consisted of three replicate aquaria, with 20 shrimps per aquarium, totaling 60 shrimps per condition and 420 shrimps across all seven groups. The experimental groups included the following:

- I: Control group (no exposure);
- II: Heavy metal cocktails (HMCs) at 0.5 mg/L;
- III: NPs50 + HMC, consisting of 50 µg/L NPs combined with 0.5 mg/L HMC;
- IV: NPs100 + HMC, with 100 µg/L NPs and 0.5 mg/L HMC;
- V: NPs150 + HMC, containing 150 µg/L NPs and 0.5 mg/L HMC;
- VI: NPs200 + HMC, which included 200 µg/L NPs and 0.5 mg/L HMC;
- VII: NPs250 + HMC, with 250 µg/L NPs and 0.5 mg/L HMC.

During the experiment, shrimp were housed in 60 L glass aquariums containing 30 L of exposure solution, with 70% water exchange daily to maintain water quality. Shrimp were exposed to the respective treatments for 14 days. Dissolved oxygen, temperature, and pH were monitored daily using a WTW-3500i multi-meter (Weilheim, Germany) to ensure stable experimental conditions.

### 2.5. Shrimp Sampling

At the end of the exposure period, all shrimp from each replicate were anesthetized on ice and sampled. Subsequently, ten shrimp from each aquarium (30 from each treatment) were dissected, and the carapace was removed. Next, the hepatopancreas was carefully separated from other tissues and placed into sterile microtubules. The hepatopancreas tissue was collected for biochemical and oxidative stress analyses following standard protocols. A total of six samples were pooled together and homogenized with a micro-homogenizer. The samples were weighed and aliquoted into two parts. These samples were placed in ice-cold phosphate-buffered saline (PBS). Next, they were homogenized and centrifuged at 12,000× *g* for 15 min at 4 °C to separate the supernatant. Finally, the supernatant was collected and stored at −26 °C for one week until biochemical and oxidative biomarker analysis.

Moreover, the muscle and hepatopancreas tissues were sampled from the remaining shrimp after exoskeleton removal. Each six samples was pooled and homogenized by a micro-homogenizer. Next, the homogenized samples were divided into two aliquots. These samples were used to measure the bioaccumulation of heavy metals and metabolic profiles. The samples were stored in liquid nitrogen for further analysis.

### 2.6. Biochemical and Antioxidant Biomarkers

Superoxide dismutase (SOD) activity was measured using the xanthine oxidase method, in which superoxide radicals generated from xanthine oxidation reduce nitro blue tetrazolium (NBT) to formazan. The degree of inhibition of formazan formation at 550 nm quantifies SOD activity, with one unit defined as 50% inhibition. Glutathione peroxidase (GPx) activity was assessed via NADPH consumption during H_2_O_2_ reduction, with absorbance measured at 340 nm. Glutathione reductase (GR) activity was estimated based on the NADPH-dependent conversion of glutathione disulfide (GSSG) to reduced glutathione (GSH), monitored at 405 nm. Catalase (CAT) activity was evaluated by the decomposition of H_2_O_2_, measured at 540 nm. GSH levels were determined using Ellman’s reagent (DTNB, 5,5′-Dithiobis(2-nitrobenzoic acid)), forming a yellow compound measured at 405 nm. Total antioxidant capacity (TAC) was assessed using the CUPRAC method, where cupric ions (Cu^2+^) are reduced to cuprous ions (Cu^+^) and react with neocuproine to form a colored complex, quantified at 450 nm. Malondialdehyde (MDA) levels, a marker of lipid peroxidation, were determined using the thiobarbituric acid reactive substances (TBARS) assay, with absorbance read at 532 nm. These oxidative stress biomarkers were measured following standard procedures, as described in Andreescu et al. [35], and reagents were provided by Kiazist Co. (Hamadan, Iran). Analyses were performed using an ELISA reader (Dana-3200 model, Garni Medical Engineering Co., Tehran, Iran), at controlled room temperature. Results were expressed as units per milligram of protein (U/mg protein) for enzymatic activities and as micromoles per gram of tissue (μmol/g tissue) for non-enzymatic antioxidants and oxidative stress markers. Protein concentration in tissue homogenates was determined using the Bradford method, with bovine serum albumin (BSA) as standard [35].

Aspartate aminotransferase (AST) activity was evaluated by monitoring the conversion of aspartate to oxaloacetate and the associated reduction in NADH at 340 nm. Alanine aminotransferase (ALT) activity was measured through the conversion of alanine to pyruvate at the same wavelength. Gamma-glutamyl transaminase (GGT) activity was determined using gamma-glutamyl-p-nitroanilide and glycylglycine substrates, with absorbance measured at 405 nm. Alkaline phosphatase (ALP) activity was assessed via hydrolysis of p-nitrophenylphosphate, monitored at 405 nm. Lactate dehydrogenase (LDH) activity was quantified using pyruvate and NADPH at 340 nm. Total protein concentration was measured with copper sulfate at 546 nm. Triglyceride levels were determined through a series of enzyme-catalyzed reactions forming a colored product, measured at 540 nm. Cholesterol was quantified using the CHOD/POD method, based on reactions catalyzed by cholesterol esterase and cholesterol oxidase, also measured at 540 nm. Glycogen levels were assessed after hydrolysis to glucose and subsequent quantification by the glucose oxidase–peroxidase method at 505 nm.

All biochemical assays were performed using Biorex-Fars Co. reagents (Shiraz, Iran), in accordance with established protocols and measured using a Unico 2100 UV/Vis spectrophotometer, provided by United Products & Instruments, Inc., (Dayton, NJ, USA). Assays were carried out at room temperature, and absorbance readings were taken immediately after reagent addition to ensure kinetic accuracy. The results were expressed as units per gram of protein (U/mg protein) for enzyme activities and as milligrams per gram of tissue (mg/g tissue) for metabolite concentrations. Methodological details for these clinical chemistry parameters are consistent with the approaches outlined in the Tietz Textbook of Clinical Chemistry and Molecular Diagnostics [36].

### 2.7. Metabolic Profile Analysis

Metabolic profiling of shrimp was conducted by homogenizing six individuals from each treatment group separately. The samples were then freeze-dried using the freeze-drier (Alpha 2.4 LD Plus, Christ Co., Osterode am Harz, Germany), ground, and passed through a 100-mesh sieve for uniformity. Whole-body metabolic analysis was performed with a confocal Raman spectrometer (Lab Ram HR, Horiba, Japan). Raman spectra were collected within the 250–1800 cm^−1^ range using a 532 nm laser at 100 mW power (GLM-532-100-SLM, Frankfurt Laser Company, Frankfurt am Main, Germany), with an integration time of 10 s and two accumulations [35,37].

### 2.8. Bio-Concentrations of Heavy Metals Analysis

The hepatopancreas samples were digested in a concentrated acid mixture containing 1 mL of 0.1 N HNO_3_, 1 mL of 0.1 N HCl, and 5 mL of 37% H_2_O_2_ using a digestion block at 90 °C. The digested solution was then diluted with deionized water to 25 mL for analysis using inductively coupled plasma mass spectrometry (ICP-MS PerkinElmer Optima 8300, PerkinElmer, Inc., Waltham, MA, USA). The diluted samples were filtered before analysis to eliminate suspended particles or undissolved solids. ICP-MS was used for trace element quantification, with argon gas creating the plasma environment, and calibration standards and quality control samples were prepared. This investigation focused on the quantification of specific elements within shrimp, including copper (Cu), iron (Fe), cadmium (Cd), lead (Pb), zinc (Zn), vanadium (V), magnesium (Mg), manganese (Mn), nickel (Ni), and cobalt (Co). The Limits of Detection (LOD) were as follows: 0.1 µg L^−1^ for Cu, Mg, and Mn; 0.05 µg L^−1^ for Pb and Cd; 1.0 µg L^−1^ for Fe; and 2.0 µg L^−1^ for Zn, 0.01 µg L^−1^ for V, Ni, and Co. Results were expressed µg/g wet tissue.

### 2.9. Integrated Biomarker Response (IBR) Calculation

The Integrated Biomarker Response (IBR) index was estimated to assess the cumulative stress response in shrimp. Biomarker data were standardized, and the IBR was calculated as described by Beliaeff and Burgeot [37].

### 2.10. Statistical Analysis

Normality of distribution was assessed using the Shapiro–Wilk test, and homogeneity of variances was evaluated with Levene’s test. Data were analyzed using IBM SPSS 24. One-way ANOVA followed by Tukey’s HSD was performed to determine significant differences among groups (*p* < 0.05). All values are expressed as mean ± standard deviation (SD). Graphical representation of the data was performed using GraphPad Prism version 8.0 to illustrate group comparisons and trends.

## 3. Results

SOD activity was significantly increased in shrimp exposed to HMC alone and combined with different concentrations of NPs. The highest activity of SOD was observed in the hepatopancreas of shrimp exposed to 200 and 250 µg/L NPS combined with 0.5 mg/L HMC. Results showed that exposure to HMC alone and combined with different concentrations of NPS significantly increased GPx activity in shrimp’s hepatopancreas. The highest activity of GPx was observed in shrimp exposed to 50 and 100 µg/L NPs combined with 0.5 mg/L HMC.

**Figure 1:**
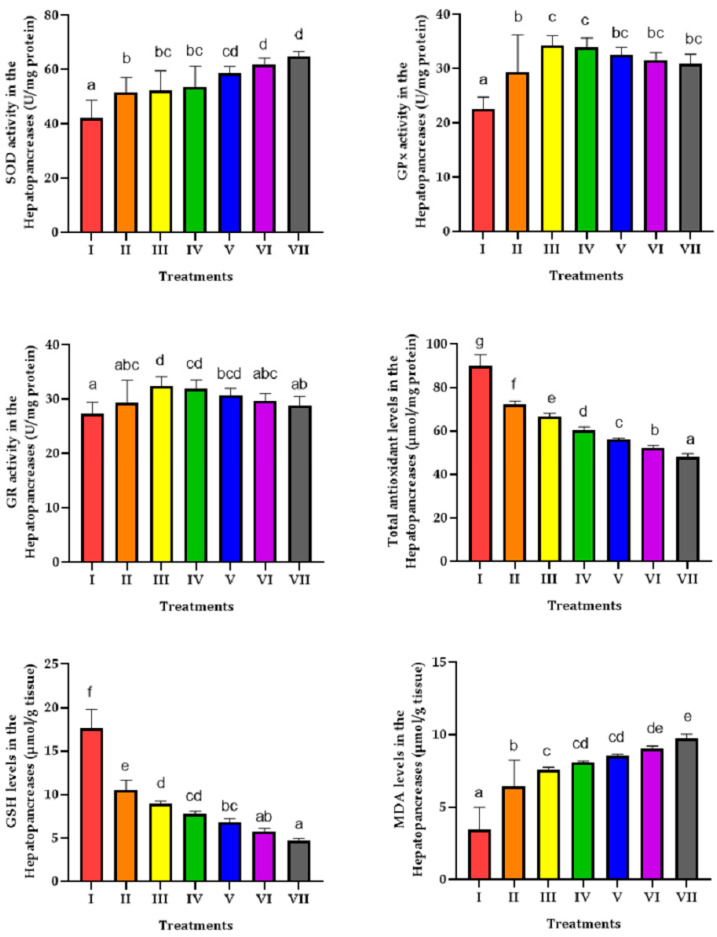
Changes in the oxidative biomarkers in the hepatopancreas of shrimp co-exposed to HMC and NPs. Different letters indicate the presence of significant differences and similar letters indicate that there is no significant difference between different experimental groups (*p* < 0.05).

The activity of GR in the hepatopancreas of shrimp exposed to 50 and 100 µg/L NPs combined with 0.5 mg/L HMC was significantly higher than in the control group. A significant decrease was observed in the cellular total antioxidant capacity and GSH contents in the hepatopancreas of shrimp exposed to HMC alone and combined with different concentrations of NPS. Exposure to HMC alone and combined with different concentrations of NPs significantly increased MDA levels in the hepatopancreas of shrimp. The findings showed that the MDA levels in the shrimp exposed to the mixture of HMC and NPs were considerably higher than in the MDA levels in the hepatopancreas of shrimp exposed to HMC alone (Figure 1).

**Figure 2:**
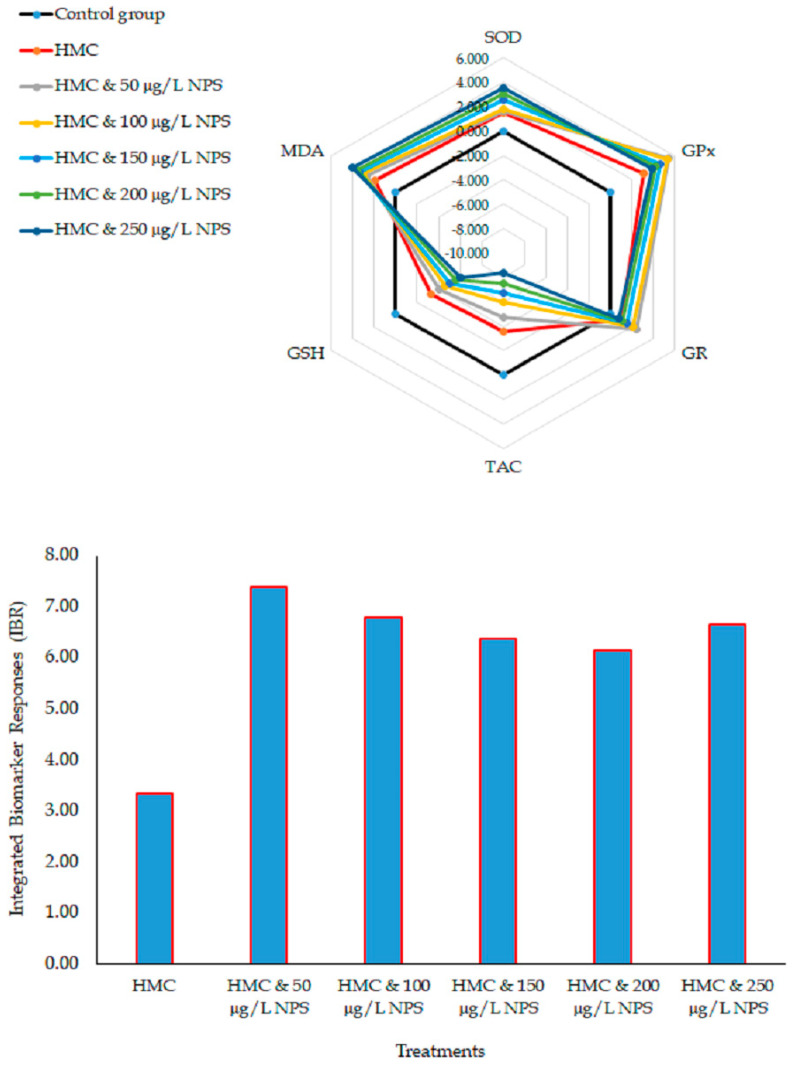
Integrated biomarker response in the hepatopancreas of shrimp co-exposed to HMC and NPs.

**Figure 3:**
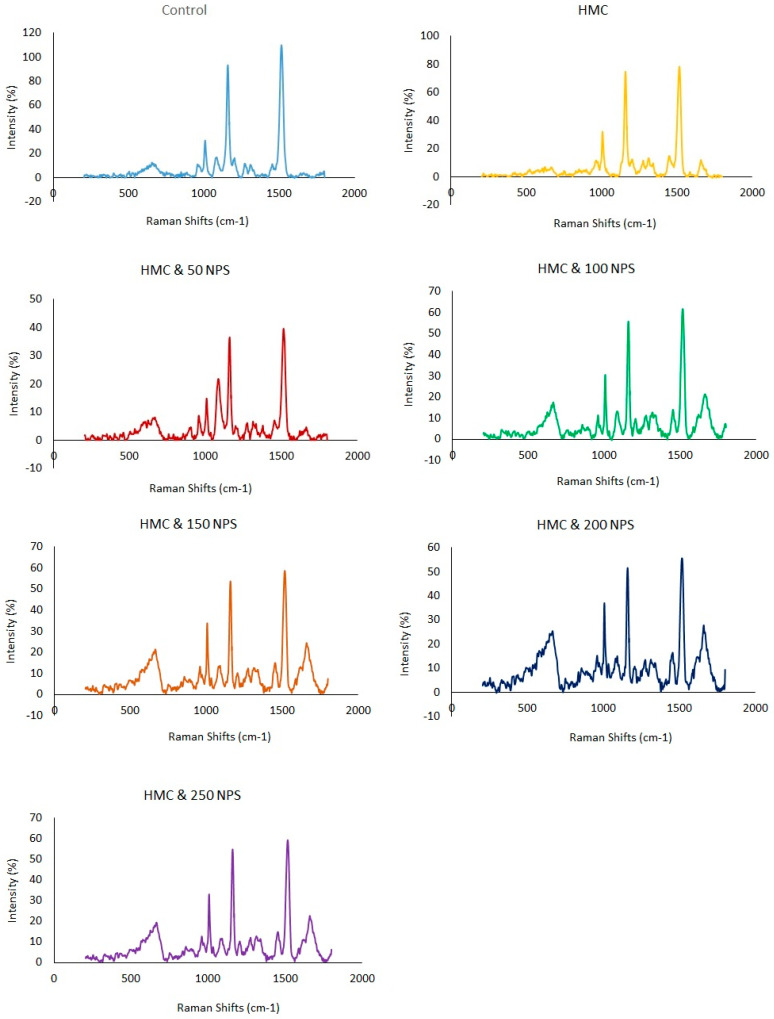
Raman spectroscopic analysis of the bio-molecular damage in shrimp exposed to HMC and NPs.

Most significant changes were observed in GPx, SOD, and GR activities and MDA levels in the hepatopancreas of shrimp following exposure to HMC and different concentrations of NPs (Figure 2). The integrated biomarker response showed that shrimp’s hepatopancreas could be more susceptible to oxidative stress after co-exposure to HMC and NPs. Integrated biomarker responses showed that co-exposure to HMC and different concentrations of NPs could induce oxidative stress in freshwater shrimp (Figure 3).

A significant increase was in the iron, zinc, cobalt, manganese, cadmium, and nickel in the freshwater shrimp co-exposed to 0.5 mg/L HMC and different concentrations of NPs. Exposure to 0.5 mg/L HMC combined with 100 µg/L and more significantly increased the bioaccumulation of copper, magnesium, vanadium, and lead in the whole body of freshwater shrimp. However, no significant differences were observed in the bioaccumulation levels of various metals between shrimp exposed to 0.5 mg/L HMC and the control group (Table 1).

Results showed that exposure to HMC alone and combined with different concentrations of NPS significantly decreased AST, GGT, and ALP activities in the hepatopancreas of shrimp. However, LDH activity was increased considerably in the hepatopancreas of shrimp exposed to HMC alone and combined with different concentrations of NPs. No significant changes in the activity of ALT. A significant decrease was observed in glycogen and total protein levels in the hepatopancreas of shrimp exposed to HMC alone and combined with different concentrations of NPs. The lowest glycogen and total protein contents were measured in the hepatopancreas of shrimp exposed to the mixture of 0.5 mg/L HMC and 250 µg/L NPs. Although no significant changes were observed in cholesterol levels in the hepatopancreas of shrimp in different groups, exposure to HMC alone and combined with different concentrations of NPS decreased triglyceride levels (Table 2).

The Raman shifts correspond to various biochemical compounds, primarily proteins, lipids, nucleic acids, and carotenoids (Figure 3). The 533 cm^−1^ peak is associated with phospholipid vibrations (PO_4_^3−^ bending) and S-S stretching in disulfide bonds of proteins. The 665 cm^−1^ shift corresponds to C-S stretching in cysteine, phosphate vibrations in nucleic acids, and ring deformations in tyrosine. The 902 cm^−1^ peak is linked to C-C stretching in polysaccharides and proteins, particularly in α-helix structures. In comparison, the 955 cm^−1^ peak is associated with C-C stretching in proteins and phosphate vibrations in nucleic acids. The 1006 cm^−1^ shift is a strong marker for phenylalanine (ring breathing mode) in proteins. The 1083 cm^−1^ peak corresponds to C-C and C-O stretching in lipids, carbohydrates, and phosphate groups found in nucleic acids and phospholipids. The 1157 cm^−1^ peak is related to C-C and C-N stretching in proteins and is also observed in carotenoids. The 1273 cm^−1^ and 1311 cm^−1^ peaks are linked to amide III vibrations (C-N stretching, N-H bending) in proteins, lipid CH_2_ wagging, and nucleic acid backbone vibrations. The 1376 cm^−1^ peak is associated with CH_3_ bending in proteins, lipids, and nucleic acids, often observed in collagen and carotenoids. The 1513 cm^−1^ shift is a characteristic carotenoid peak (C=C stretching in conjugated polyenes) and is also linked to aromatic amino acids like tryptophan. The 1620 cm^−1^ peak corresponds to C=C stretching in aromatic amino acids such as tyrosine, tryptophan, and phenylalanine, as well as secondary structures of proteins (amide I band). Finally, the 1662 cm^−1^ shift is associated with amide I vibrations (C=O stretching in proteins), indicative of α-helical or β-sheet secondary structures. Changes in Raman shifts after shrimp exposure to HMC and NPS indicated biochemical alterations. Shifts in bands of amide I (1662 cm^−1^) and amide III (1273, 1311 cm^−1^) suggested protein unfolding. Alterations in phenylalanine (1006 cm^−1^), tryptophan (1513 cm^−1^), and tyrosine (1620 cm^−1^) indicated protein oxidation. Changes in the 533 and 665 cm^−1^ peaks may be related to membrane damage and disulfide bond disruption. Results showed that alterations in 1083 and 1376 cm^−1^ may be reflected in lipid peroxidation. Furthermore, shifts at 902 and 955 cm^−1^ indicated damage to DNA and RNA. These shifts showed oxidative stress, bio-molecular degradation, and metabolic disturbances (Figure 3).

## 4. Discussion

Studies on aquatic environments have demonstrated that numerous pollutants can significantly impact both freshwater and marine organisms [38,39]. In particular, the interactions between different xenobiotics in water create a complex network of effects that influence their bioavailability and toxicity [40]. Understanding these interactions is crucial, as contaminants such as nanoplastics and heavy metals may not only exert individual toxic effects, but NPS can modulate HMC behavior in aquatic ecosystems.

Our results showed that co-exposure to NPs and HMCs significantly increased the bioaccumulation of several metals in shrimp tissues, particularly at higher concentrations of NPs. These results support the hypothesis that NPs may act as carriers, enhancing the uptake of heavy metals through adsorption and increased tissue permeability.

In light of this evidence, the discussion first addresses the mechanisms and patterns of heavy metal bioaccumulation observed in the various treatments, as this process is probably one of the main factors in the physiological changes recorded. Then, the interaction between NPs and HMCs contributes to oxidative stress, enzymatic alterations, and metabolic changes are also discussed, highlighting potential synergistic effects.

In this context, *M. rosenbergii* may absorb trace elements and heavy metals through several primary routes, including the gills, which serve as the main pathway for metal uptake from surrounding water. Metals can also enter via the digestive tract when the organism ingests contaminated food or sediment, and to a lesser extent, through the exoskeleton during molting, where limited passive diffusion may occur [41]. At the cellular level, some metals can enter cells through passive diffusion. This is especially true for lipophilic types, such as methylmercury, which can easily cross cell membranes without needing special transporters. This process depends on the metal’s concentration in the environment. Alternatively, metals that mimic essential ions, such as Cd^2+^ mimicking Ca^2+^ or Zn^2+^, may utilize specific ion channels and transporters [42]. Examples include calcium channels that can transport Cd^2+^ and Pb^2+^, iron transporters like DMT1 involved in Mn^2+^, Fe^2+^, and Cd^2+^ uptake, and copper transporters such as CTR1 for Cu and Ag. Some metals bound to particles or organic ligands may also be internalized through endocytosis, especially in the epithelial cells of the gills and hepatopancreas [43]. On a molecular level, crustaceans employ various mechanisms to manage metal accumulation and toxicity [41]. Metallothioneins (MTs) are low-molecular-weight, cysteine-rich proteins that bind metals with high affinity, particularly Cd, Cu, and Zn, playing key roles in detoxification and maintaining metal homeostasis [44]. Metal exposure activates their expression via metal-responsive transcription factor 1 (MTF-1) [45]. Heat shock proteins (HSPs) are also induced under metal stress, assisting in protein folding and preventing the aggregation of damaged proteins [46]. ATP-binding cassette (ABC) transporters, such as P-glycoprotein (P-gp) and multidrug resistance-associated proteins (MRPs), facilitate the expulsion of toxicants from cells [47]. Moreover, glutathione (GSH)-based systems conjugate with electrophilic metal species, aiding their excretion via glutathione S-transferases (GSTs) and MRPs. Heavy metal exposure often leads to the production of ROS, prompting the activation of enzymatic antioxidant defenses, including SOD, CAT, and GPx, which collectively mitigate oxidative damage [48]. At the molecular level, exposure to both NPs and heavy metals can significantly enhance the expression of key stress response genes and detoxification proteins in crustaceans [49]. MTs, which bind metals to aid in detoxification and homeostasis, are often elevated in response to increased intracellular metal concentrations, with their expression typically amplified when NPs and MPs are present [50]. Fan et al. [51] showed that to counteract increased ROS production, antioxidant enzymes like SOD, CAT, GPx, and GST are upregulated. Additionally, ATP-binding cassette (ABC) transporters, including P-glycoprotein (P-gp) and multidrug resistance-associated proteins (MRPs), are activated to help expel toxic substances from cells [52]. However, their effectiveness can decrease when these defense systems are overwhelmed by the combined presence of NPs and heavy metals. Studies have shown that NPs may interfere with gene regulation pathways, such as those involving Nrf2, which controls antioxidant responses, and MTF-1, involved in MT induction [53].

The findings from the present study showed that freshwater shrimp exposed to HMC did not show significant changes in Cu, Mg, Co, Mn, V, and Cd concentrations. The results showed that under normal conditions, shrimp may rely on efficient cellular and molecular mechanisms, such as metal-binding proteins (e.g., metallothioneins), ion transporters, and detoxification pathways, to maintain metal homeostasis and prevent excessive metal accumulation. However, co-exposure to HMC and NPs led to a significant increase in the bioaccumulation of metals. Therefore, NPs could affect the bioaccumulation rate of metals in the body of freshwater shrimp acting as carriers and facilitating metal entry through uptake or endocytosis. NPs can also potentially damage cellular barriers, such as the gill or hepatopancreas epithelium, thereby increasing tissue permeability. Furthermore, oxidative stress and inflammation induced by NPs may change the expression and function of metal transporters, disrupt detoxification processes, and further contribute to metal accumulation.

Therefore, results from the present study showed a synergistic effect between HMCs and NPs. In other words, exposure of shrimp to NPs appears to be able to disrupt their ability to regulate metal uptake and excretion, ultimately increasing their sensitivity to metal toxicity at the cellular level, as also reported by Srivastav et al. [54], Yu et al. [55], Chen et al. [56], and Arif et al. [32].

As a result, a significant change in the antioxidant system in the freshwater shrimp in response to the exposure to NPs, MHC, and their mixture was demonstrated. Heavy metals are known to be able to disrupt redox homeostasis by generating ROS through redox cycling, mitochondrial dysfunction, and Fenton reactions [57]. Similarly, NPs that, due to their small size and high surface area, can penetrate cellular membranes, accumulate in organelles, such as mitochondria and lysosomes [58], may induce significant oxidative stress in shrimp by generating excessive ROS, which in turn disrupt the balance of the cellular antioxidant defense. In this study, a significant increase in SOD activity was observed in the hepatopancreas of shrimp exposed to HMC alone and in combination with NPs, suggesting an accumulation of superoxide radicals. Interestingly, this response appeared to be dose-dependent, as higher concentrations of NPs led to a progressive enhancement of SOD activity. The concomitant rise in GPx activity may represent an antioxidant defense mechanism aimed at neutralizing the increased levels of hydrogen peroxide generated by SOD activity. GPx plays a key role in detoxifying reactive oxygen species (ROS) by catalyzing their conjugation with reduced glutathione (GSH), making its upregulation a critical response to oxidative stress. This process relies on adequate GSH availability, which in turn depends on the activity of glutathione reductase (GR). The observed increase in GR activity in treated shrimp may reflect a compensatory mechanism to regenerate GSH from its oxidized form (GSSG). However, despite elevated GR activity, GSH levels were significantly reduced following exposure to HMC and NPs, suggesting that the demand for GSH exceeded the capacity for its regeneration. Since GSH recycling depends on the availability of NADPH, it is plausible that reduced GSH levels result from NADPH depletion under stress conditions. Ultimately, the marked decline in GSH could compromise the cellular antioxidant defense system, exacerbating the oxidative damage induced by co-exposure to HMC and NPs. The results from the present study could be compared with those reported in the literature [31,59,60,61,62,63,64,65].

Results from our study highlighted IBR changes, indicating a synergistic effect of NPs on the toxicity of HMC in inducing oxidative stress in shrimp. Varó et al. [66] found that *Artemia franciscana* exposed to PS-NH_2_ showed reduced antioxidant enzyme activity and lipid peroxidation over time. Zeng et al. [67] reported oxidative stress and hepatopancreatic damage in *L. vannamei* due to MPs accumulation.

Results showed a significant decrease in the cellular total antioxidant capacity in shrimp after exposure to HMC alone and combined with NPs, indicating a decrease in the ability of the cellular antioxidant defense system to neutralize ROS. In this situation, the lipid peroxidation rate would be increased in the shrimp’s hepatopancreas. A significant increase in MDA levels showed an increased lipid peroxidation rate in the hepatopancreas of shrimp exposed to HMC alone and combined with NPs. Hence, decreased cellular total antioxidant capacity and increased MDA levels indicate oxidative stress in the shrimp’s hepatopancreas. Generally, the findings highlighted the synergistic effects of higher concentrations of NPs on the toxicity of HMC. These findings displayed that combined exposure to heavy metals and NPs synergistically disrupts redox homeostasis in the hepatopancreas of shrimp, leading to enhanced oxidative stress.

The hepatopancreas of shrimp plays a critical role in metabolic functions, detoxification, and enzyme regulation. Exposure to HMC and NPs can induce oxidative stress, mitochondrial dysfunction, and membrane damage in the hepatopancreas of shrimp, as reported by Moruf et al. [68] and Yu et al. [55]. Oxidative stress could affect the stability and physiological functions of cellular membranes. A decrease in AST, ALT, GGT, and ALP activities in the hepatopancreas of shrimp co-exposed to NPs and HMC may indicate severe cellular damage and functional suppression of this organ. This reduction may result from the inhibition of enzyme activity due to the binding of metals to active sites. Damaged biological membranes can change hepatocyte membrane permeability and release cytoplasmic enzymes into the hemolymph, including AST, ALT, and GGT [69]. Thus, AST, ALT, and GGT activities would be decreased in cells. Moreover, exposure to HMC alone and combined with NPS might inhibit AST, ALT, and GGT activities in the hepatopancreas of shrimp. A significant decrease in ALP activity as a transmembrane enzyme might be related to its inactivation or biological degradation. These findings showed that the reduction may result from disrupted enzyme synthesis due to damage to protein production pathways or from extensive cell death that limits enzyme release. Additionally, mitochondrial dysfunction and energy depletion may impair overall metabolic activity. However, a significant increase in LDH indicated the creation of hypoxic conditions in the cell and an increase in the rate of cellular anaerobic metabolism [70].

Exposure to heavy metals and NPs can disrupt energy metabolism and lipid–protein homeostasis in the hepatopancreas of shrimp. These toxicants induce oxidative stress, mitochondrial dysfunction, and damage to cellular structures, leading to changed levels of key biochemical indicators. A significant decrease in glycogen levels might be related to increased energy needs to combat the toxicity effects of HMC alone and a mixture with NPS. In a stressful situation, glycogen storage in the hepatopancreas would be degraded to glucose to balance energy sources and needs. Similarly, Li et al. [71] found that NPs altered glucose metabolism in *L. vannamei*, reducing glucose and affecting hormone levels, enzymes, and gene expression, leading to metabolic disruptions.

A significant decrease in total protein levels indicated disruption in protein biosynthesis in the hepatopancreas of shrimp exposed to HMC alone and combined with different concentrations of NPS. Increased proteolysis may affect the total protein. Decreased total protein could affect the regeneration of tissues that are hurt following exposure to environmental pollutants [70].

Decreasing triglyceride contents in the hepatopancreas of shrimp exposed to HMC alone and combined with different concentrations of NPS indicated the disruption in lipid metabolism. This reduction may indicate impaired lipid synthesis, altered lipoprotein transport, or increased lipid catabolism. These effects could be due to the toxic impact of HMC on hepatopancreatic cells or the interference of NPs in normal metabolic signaling pathways, as similarly found by Faramazinia et al. [72], whose study reported that MPs and DPP exposure in goldfish resulted in elevated plasma glucose, creatinine, triglycerides, and cholesterol, along with liver damage and impaired protein synthesis, suggesting metabolic and immune dysfunction. Similarly, Nematdoost Haghi and Banaee [73] found that exposure to paraquat and microplastics in *Cyprinus carpio* led to increased AST, ALT, ALP, LDH, and CPK activities. They found that the significant biochemical disruptions could be attributed to a synergistic toxic effect. Additionally, reductions in total protein, globulin, cholesterol, and triglycerides pointed to impaired liver function and metabolic homeostasis.

Changes in these Raman shifts after shrimp were exposed to HMC combined with NPs indicated significant biochemical alterations, including disruptions in protein secondary structures, lipid peroxidation, and nucleic acid damage. Heavy metals and NPS may bind directly to nucleic acids or induce DNA and RNA strand breaks and base modifications. Moreover, they can disrupt DNA repair enzymes, leading to mutations and impaired cell functions. Shifts in the amide I (1662 cm^−1^) and amide III (1273 and 1311 cm^−1^) bands showed protein unfolding or conformational changes, possibly due to oxidative stress or direct metal–protein interactions. These changes indicated alterations in the amide I (mainly C=O stretching) and amide III (mainly N–H bending and C–N stretching) bands. These findings reflected protein unfolding, denaturation, or conformational changes. Therefore, these modifications impair protein function and stability in shrimp hepatopancreas and muscle tissues. Heavy metals and NPs may disrupt the metabolism of aromatic amino acids such as phenylalanine, tryptophan, and tyrosine by inducing oxidative stress, enzyme inhibition, and tissue damage [74]. Changes in phenylalanine (1006 cm^−1^), tryptophan (1513 cm^−1^), and tyrosine (1620 cm^−1^) signals indicated modifications in aromatic amino acids, which could reflect protein oxidation or aggregation. These toxicants can impair key enzymes involved in amino acid catabolism and biosynthetic pathways, such as phenylalanine hydroxylase and tryptophan hydroxylase, leading to altered levels of these amino acids. Additionally, oxidative damage to hepatopancreatic tissue may reduce the uptake and utilization of amino acids or increase their degradation. Therefore, changes in phenylalanine, tryptophan, and tyrosine levels reflect disrupted protein metabolism and stress-induced metabolic imbalance in shrimp. The results showed that exposure to HMC and NPs can cause oxidative damage to phospholipids in the cellular membrane. The 533 and 665 cm^−1^ peaks, associated with phospholipids and sulfur-containing amino acids, were affected, suggesting damage to membrane integrity and disulfide bond disruption. Changes in the 1083 cm^−1^ (lipid/phosphate vibrations) and 1376 cm^−1^ (CH_3_ bending in nucleic acids and lipids) peaks may indicate lipid peroxidation and possible interactions with nucleic acids. Changes in the 902 and 955 cm^−1^ (C-C and phosphate vibrations) peaks further indicated structural damage to DNA or RNA. These shifts provided evidence of oxidative stress, bio-molecular degradation, and metabolic disturbances induced by the combined toxicity of HMC and NPs in shrimp.

## 5. Conclusions

The findings of this study demonstrated that co-exposure to NPs and HMC induced oxidative stress, biochemical alterations, and bioaccumulation in *M. rosenbergii*. NPs enhance heavy metal accumulation in shrimp by acting as a carrier, delivering metals into tissues. This leads to increased toxicity, disrupted metal balance, oxidative stress, and impaired metabolism. These effects may cause cellular damage and reduce shrimp survival. The observed increase in antioxidant enzyme activities and malondialdehyde levels, coupled with a decrease in total antioxidant capacity, highlighted significant oxidative damage in the shrimp hepatopancreas. Biochemical disruptions, such as altered enzymatic activity and diminished energy reserves, further indicated a state of metabolic stress. Additionally, Raman spectroscopy analysis revealed molecular-level disturbances, including protein oxidation, membrane damage, and nucleic acid degradation. These results suggest that NPs can exacerbate heavy metal toxicity, amplifying their potential risks to the health of freshwater aquatic organisms, including *M. rosenbergii*, and posing a broader threat to aquatic ecosystems.

## Figures and Tables

**Table 1:** The bioaccumulation of heavy metals in the whole body of shrimp co-exposed to heavy metal cocktails and Nano-polyethylene.

Bioaccumulation of Heavy Metals (µg/g Tissue)	Control	0.5 mg/L HMC	0.5 mg/L HMC & 50.0 µg/L NPS	0.5 mg/L HMC & 100 µg/L NPS	0.5 mg/L HMC & 150 µg/L NPS	0.5 mg/L HMC & 200 µg/L NPS	0.5 mg/L HMC & 250 µg/L NPS
Fe	6.59 ± 1.28 ^a^	13.51 ± 1.87 ^b^	15.33 ± 3.61 ^bc^	18.33 ± 3.44 ^c^	25.67 ± 5.32 ^d^	27.83 ± 4.45 ^de^	31.83 ± 4.45 ^e^
Cu	2.49 ± 0.98 ^a^	6.50 ± 2.07 ^ab^	10.67 ± 5.24 ^bc^	13.83 ± 2.32 ^c^	21.83 ± 8.59 ^d^	34.33 ± 3.67 ^e^	37.00 ± 2.19 ^e^
Zn	4.85 ± 2.15 ^a^	11.90 ± 2.65 ^b^	13.33 ± 3.01 ^b^	15.17 ± 2.79 ^b^	22.83 ± 6.55 ^c^	26.67 ± 5.99 ^cd^	28.17 ± 4.54 ^d^
Mg	1.00 ± 0.64 ^a^	2.53 ± 1.23 ^ab^	4.62 ± 2.63 ^b^	8.00 ± 1.10 ^c^	13.00 ± 3.52 ^d^	16.67 ± 2.88 ^e^	17.50 ± 2.43 ^e^
Co	0.72 ± 0.46 ^a^	2.15 ± 0.32 ^ab^	4.33 ± 2.25 ^b^	5.50 ± 2.35 ^b^	7.67 ± 2.25 ^c^	9.00 ± 0.63 ^c^	9.50 ± 0.55 ^c^
Mn	1.76 ± 0.50 ^a^	5.00 ± 0.87 ^ab^	11.17 ± 4.49 ^b^	13.33 ± 3.01 ^c^	21.00 ± 6.99 ^d^	28.67 ± 5.16 ^e^	29.50 ± 4.09 ^e^
V	0.40 ± 0.22 ^a^	1.95 ± 0.32 ^ab^	3.33 ± 1.03 ^bc^	4.00 ± 0.63 ^bc^	5.67 ± 1.86 ^c^	11.17 ± 2.14 ^d^	11.33 ± 1.75 ^d^
Cd	0.93 ± 0.51 ^a^	7.85 ± 0.96 ^a^	23.83 ± 4.71 ^b^	29.33 ± 5.28 ^b^	43.00 ± 12.41 ^c^	46.00 ± 8.99 ^cd^	53.50 ± 9.01 ^d^
Ni	0.56 ± 0.42 ^a^	5.53 ± 1.12 ^b^	7.50 ± 2.26 ^b^	7.67 ± 1.75 ^b^	11.83 ± 4.26 ^c^	16.50 ± 2.59 ^d^	18.17 ± 2.23 ^d^
Pb	0.71 ± 0.45 ^a^	5.05 ± 2.14 ^b^	8.00 ± 2.90 ^bc^	9.17 ± 1.72 ^c^	15.83 ± 6.24 ^d^	19.00 ± 3.03 ^de^	21.50 ± 2.95 ^e^

Different letters indicate the presence of significant differences and similar letters indicate that there is no significant difference between different experimental groups (*p* < 0.05).

**Table 2:** Changes in the biochemical parameters in the hepatopancreas of shrimp co-exposed to heavy metal cocktails and nano-polyethylene.

Biochemical Parameters	Control	0.5 mg/L HMC	0.5 mg/L HMC & 50.0 µg/L NPS	0.5 mg/L HMC & 100 µg/L NPS	0.5 mg/L HMC & 150 µg/L NPS	0.5 mg/L HMC & 200 µg/L NPS	0.5 mg/L HMC & 250 µg/L NPS
(U/g protein)							
AST	8.8 ± 1.4 ^b^	5.6 ± 2 ^a^	5.3 ± 1.9 ^a^	5.1 ± 1.8 ^a^	4.9 ± 1.6 ^a^	4.7 ± 1.5 ^a^	4.4 ± 1.5 ^a^
ALT	5 ± 1.7 ^a^	3.2 ± 1.4 ^a^	3.1 ± 1.3 ^a^	3.0 ± 1.2 ^a^	2.9 ± 1.1 ^a^	2.7 ± 1.1 ^a^	2.6 ± 1.0 ^a^
ALP	12.2 ± 2.4 ^b^	8.8 ± 2.0 ^ab^	8.3 ± 2.0 ^a^	7.9 ± 1.8 ^a^	7.6 ± 1.7 ^a^	7.3 ± 1.6 ^a^	6.9 ± 1.5 ^a^
GGT	3.8 ± 1.2 ^b^	2.2 ± 0.3 ^a^	1.9 ± 0.4 ^a^	1.8 ± 0.5 ^a^	1.9 ± 0.4 ^a^	1.7 ± 0.4 ^a^	1.7 ± 0.4 ^a^
LDH	32.8 ± 1.9 ^a^	56.3 ± 11.8 ^ab^	57.7 ± 12.2 ^b^	56.0 ± 11.8 ^ab^	59.6 ± 15.0 ^b^	58.1 ± 14.7 ^b^	56.8 ± 14.4 ^b^
(mg/g tissue)							
Glycogen	31.3 ± 5.2 ^b^	22.8 ± 4.5 ^a^	21.7 ± 4.3 ^a^	20.5 ± 4.1 ^a^	19.3 ± 3.9 ^a^	18.3 ± 3.9 ^a^	17.2 ± 3.7 ^a^
Cholesterol	26.5 ± 9.6 ^a^	25.2 ± 7.8 ^a^	20.5 ± 5.2 ^a^	19.3 ± 5.0 ^a^	18.2 ± 4.7 ^a^	17.6 ± 4.7 ^a^	16.8 ± 4.5 ^a^
Triglycerides	43.2 ± 0.51 ^b^	38.3 ± 0.96 ^ab^	32.8 ± 4.71 ^ab^	31.2 ± 5.28 ^ab^	29.5 ± 12.41 ^a^	27.8 ± 8.99 ^a^	30.3 ± 9.01 ^a^
Protein	4.5 ± 0.6 ^c^	4.0 ± 0.4 ^bc^	3.8 ± 0.4 ^abc^	3.6 ± 0.3 ^ab^	3.5 ± 0.3 ^ab^	3.3 ± 0.2 ^a^	3.2 ± 0.2 ^a^

Different letters indicate the presence of significant differences and similar letters indicate that there is no significant difference between different experimental groups (*p* < 0.05).

## Data Availability

The original contributions presented in this study are included in this article. Further inquiries can be directed to the corresponding authors.
